# Do Clinicians Ask Pregnant Women about Exposures to Tobacco and Cannabis Smoking, Second-Hand-Smoke and E-Cigarettes? An Australian National Cross-Sectional Survey

**DOI:** 10.3390/ijerph14121585

**Published:** 2017-12-16

**Authors:** Gillian S. Gould, Yael Bar Zeev, Laura Tywman, Christopher Oldmeadow, Simon Chiu, Marilyn Clarke, Billie Bonevski

**Affiliations:** 1 School of Medicine and Public Health, The University of Newcastle, University Drive, Callaghan, NSW 2308, Australia; Yael.BarZeev@uon.edu.au (Y.B.Z.); laura.twyman@nswcc.org.au (L.T.); billie.bonevski@newcastle.edu.au (B.B.); 2 Hunter Medical Research Institute, Newcastle, NSW 2305, Australia; christopher.oldmeadow@hmri.org.au (C.O.); simon.chiu@hmri.org.au (S.C.); 3 Clarence Specialist Clinic, Grafton, NSW 2460, Australia; marilyn.clarke@clarencespecialistclinic.com.au

**Keywords:** pregnancy, electronic cigarettes, cannabis, tobacco, smoking, tobacco, smokeless tobacco

## Abstract

Clinicians often ask pregnant women about tobacco smoking, but their practices of asking about other smoking and nicotine exposures are unknown. This study analysed how often clinicians ask pregnant women about their use of e-cigarettes, cannabis, chewing tobacco, and second-hand smoke (SHS) exposure. Two cross-sectional surveys were undertaken. A random sample of 500 General Practitioner (GP) members were invited from the National Faculty of Aboriginal and Torres Strait Islander Health (NFATSIH) to complete an on-line survey, and 5571 GP and Obstetrician (OBS) members of the Royal Australian and New Zealand College of Obstetricians and Gynaecologists (RANZCOG) were sent a paper survey by mail. Questions on frequency of asking about the exposures used Likert Scales, later dichotomized to “often-always” and “never-sometimes”. Logistic regressions estimated associations between clinician type and asking about cannabis, e-cigarettes, chewing tobacco, and SHS. An adjusted model reduced potential confounders of location, guidelines, gender and population. *n* = 378 GPs and OBS participated (6.2% response). In total, 13–14% asked “often-always” about e-cigarettes; 58% cannabis; 38% cannabis with tobacco; 27% SHS, and 10% chewing tobacco—compared to 95% of the sample asking about cigarette smoking. After adjustment, the odds of RANZCOG GPs (OR 0.34) and OBS (OR 0.63) asking about cannabis were lower compared to NFATSIH GPs. Clinician type was non-significant for asking about e-cigarettes, chewing tobacco and SHS. Surveyed Australian GPs and obstetricians asked less frequently about e-cigarettes, chewing, SHS exposure, and cannabis, potentially missing important exposures for mother and child.

## 1. Introduction 

Early exposures of the foetus to a range of toxic substances can adversely influence the life trajectory. Tobacco smoking has well-known major adverse effects on the developing foetus and also the mother [1]. For the foetus, these influences from tobacco may include growth restriction, pre-term birth, stillbirth, abnormal lung and neurological development, and birth defects. If a mother smokes during pregnancy, her child is more likely to experience respiratory illnesses, childhood cancer, learning and behavioural problems, and chronic diseases such as obesity, respiratory, cardiac, and diabetes [1].

The prevalence of tobacco smoking among Australian pregnant women has been reducing in the general population, from 15% in 2009, to 12% in 2013 [2]. Slower rates of decline are reported globally in women in high priority groups and those experiencing health disparities [3]. In Australia, smoking prevalence remains higher among pregnant women from low socio-economic postcodes (20%), geographically remote locations (37%), youth (34%), and Aboriginal and Torres Strait Islander women (47%) [2].

Over 75% of health professionals in Australia are reliably asking pregnant women about their tobacco or cigarette smoking, and recording their smoking status [4,5]. However, pregnant women may be exposed to other types of smoking (such as cannabis), tobacco in other forms (smokeless tobacco), and some newer exposures to nicotine have appeared (e-cigarettes). The practices of health professionals may need to accommodate the advent of emerging and under-recognised exposures during pregnancy, along with other traditional exposures. These exposures may be country-specific such as snus (mucosal absorption of tobacco) in Sweden, waterpipe smoking (hookah) in the Middle East, and chewing tobacco in India. Aboriginal and Torres Strait Islanders in some rural or remote communities may chew block tobacco or native tobacco (pituri). However, some of these practices have become popular with youth outside of these countries. These other exposures have also been associated with low birth weight [6,7,8], and other adverse effects such as pre-term birth and stillbirth [8]. 

E-cigarette use, so far, is reported infrequently during pregnancy [9,10]. There are no surveillance figures to indicate the prevalence of e-cigarette smoking in pregnant women. Of 316 pregnant women surveyed through a US university clinic, 43% perceived e-cigarettes to be less harmful to the foetus than smoking [11]. This perception may induce pregnant women to use e-cigarettes more freely or to switch to vaping once becoming pregnant [12]. However, the nicotine delivered through e-cigarettes may not be innocuous for foetal lung development in humans [13,14]. There is also the potential for toxic exposure through e-cigarette aerosols according to mouse models [15]. This needs to be balanced with the likelihood that smoking e-cigarettes in pregnancy may be less dangerous for the mother [16].

Smoking cannabis (commonly mixed with tobacco) is reported in 2–3% of Australian women in general when pregnant [17,18], and in 15–20.5% of Aboriginal and Torres Strait Islander counterparts [19,20]. Persistent use of cannabis in pregnancy can cause growth restriction [17]. Babies born to mothers who are using cannabis, are more likely to need neonatal intensive care [21]. 

Exposure to second-hand smoke (SHS), can have adverse effects on the growing foetus, and is associated with pregnancy complications [22,23]. The number of smokers in the household impacts a pregnant woman’s level of exposure to SHS [24]. Partner smoking is a particular concern as the genetic effects from tobacco may pre-date conception [25]. Exposure to SHS is reducing in most populations, however women may be at more risk within high prevalence populations.

In light of the potentially serious nature of these effects, it has been suggested that all types of smoking, nicotine and tobacco exposures are recorded and assessed [26]. Several studies in Australia have focused on the overall practices of health professionals in asking and assessing pregnant women for tobacco smoking, and the type of care delivered to them [27]. However no study, as far as we know, has focused on how often Australian clinicians ask or screen pregnant women about other types of smoking or nicotine-related exposures such as e-cigarettes, tobacco in other forms (potentially with cannabis or chewed), or exposure to SHS.

This study therefore had two aims: to survey Australian GPs and obstetricians about: (a) their practices of asking pregnant women about their use of e-cigarettes, forms of tobacco use other than regular cigarettes, cannabis use, and exposure to SHS, and (b) to compare differences in the responses of different clinicians’ groups. Practice location (rurality), and practice constituency (general population, or higher proportion of Aboriginal population) was hypothesised to have an impact on these clinician practices, because prevalence in rural and Aboriginal and Torres Strait Islander populations is usually higher that of the general or urban population.

## 2. Materials and Methods

### 2.1. Design

The study was a self-administered national cross-sectional survey that included two sampling strategies. This questionnaire forms a subset of questions in the full survey previously published as supplementary material [5].

### 2.2. Sample

- An on-line survey of a random sample of 500 GPs from the National Faculty of Aboriginal and Torres Strait Islander Health (NFATSIH) database of the Royal Australian College of General Practitioners (RACGP). The randomisation was achieved by requesting the RACGP to select the first 500 names after they shuffled the database names on an Excel spreadsheet. This population was chosen as they are GPs who are interested, actively engaged or have past experience in Aboriginal and Torres Strait Islander Health; and/or identify as an Aboriginal or Torres Strait Islander GP. This sample was relevant as in Australia, Aboriginal and Torres Strait Islander women have a higher prevalence of tobacco smoking than the general population, and thus more likely to be exposed, for example, to SHS.
- A paper-based survey of GPs and Obstetricians from the complete mail-out list of 5571 members of the Royal Australian and New Zealand College of Obstetricians and Gynaecologists (RANZCOG). This group included fellows and diplomates of the college and GPs and Obstetricians in training.

All participants were required to be in active practice (not retired or students) and consult with pregnant women.

### 2.3. Procedures

A link to the survey form was sent by email to the NFATSIH GPs from the RACGP administration, and followed up with one reminder, as per Faculty policy. 

The RANZCOG members were sent the paper survey as a printed insert in their “O&G” magazine. The respondent had an option to post or fax back the completed form. No reminders were feasible.

The study was approved by the University of Newcastle Human Research Ethics Committee on 18 March 2015, approval number H-2015-0067.

### 2.4. Measures

#### 2.4.1. Participant Characteristics

Both surveys included demographic questions: gender (male or female), smoking status (daily, occasional, ex-smoker, never smoked), years since medical qualification (less than 10 years, 10–19 years, 20 plus years), constituency of practice population (mainly for general population, mainly for Aboriginal or Torres Strait Islander population, or other) and postcode. The on-line survey asked respondents to indicate the Remoteness Area classification of their main practice location (asked to select R1–R5) [28], whereas the paper survey asked “Please enter the postcode of your main work location”. Respondents were asked if they had read any from a list of 5 smoking cessation guidelines (yes or no). 

#### 2.4.2. Screening for Substance Use and Exposure

Participants were asked about their practices of asking about the following exposures: *How often do you ask a pregnant client about using these substances?* (Options on a 5-point Likert scale: Never, Occasionally, Sometimes, Often, or Always.)

- Cannabis
- Cannabis with tobacco
- E-cigarettes with nicotine
- E-cigarettes without nicotine
- Chewing tobacco
- Second hand smoke (SHS) exposure

### 2.5. Analysis

Data were analysed with IBM SPSS v24 (IBM Corp, Armonk, NY, USA) and R 3.3.2. (CRAN, Vienna, Austria). Descriptive analyses of variables used counts and percentages for categorical measures for all respondents together, and the three groups separately (NFATSIH GPs, RANZCOG GPs and RANZCOG Obstetricians). 

#### 2.5.1. Transformation of Variables

Australian Standard Geographical Classification Remoteness Area classification of the clinician’s main practice location was calculated from the postcode. Geographical location of the practices was collapsed into three categories: (urban—RA1; regional—RA 2 plus 3; remote—RA 4 plus 5). Guideline use was transformed in a variable “read any guideline”.

Clinicians’ responses from the Likert Scales above were collapsed into two categories “Never, Occasional, Sometimes” and “Often, Always”. 

Population constituency (general population versus >30% Aboriginal or Torres Strait Islander population) was calculated from those in the survey who indicated their practice was “mainly for the Aboriginal or Torres Strait Islander population” combined with those who selected “other” and commented that the practice catered for 30% or more Aboriginal or Torres Strait Islander people, versus the rest (general population).

#### 2.5.2. Logistic Regression

Logistic regression models were used to estimate odds ratios (OR) for the association between the type of health professional (RANZCOG OBS; RANZCOG GP; NFATSIH GP) and enquiry about each of cannabis, e-cigarettes (with nicotine), chewing tobacco, and exposure to SHS.

Further adjustments to the OR were calculated to reduce the potentially confounding effects of practice location, reading of clinical guidelines, clinician’s gender and population constituency, due to potential associations with both ‘exposure’ and ‘outcome’. For example, rural and Aboriginal or Torres Strait Islander women are more likely to be subject to SHS exposure, thus their clinicians may also remember to ask about these exposures more often. Variance Inflated Factors (VIF) were investigated to check for multicollinearity. Crude and adjusted ORs for the effect of clinician type (NFATSIH GP vs RANZCOG GP and RANZCOG OBS) are presented together with 95% confidence intervals and *p*-values. Parameter estimates for confounding variables are not presented.

## 3. Results

In total, *n* = 378 participants were recruited. These comprised 42 GPs from the NFATSIH on-line survey (response rate 8.4%); 157 GPs from the RANZCOG paper survey, and 178 Obstetricians from the RANZCOG paper survey (response rate 6%).

Characteristics of the sample are in Table 1. Participants came from all states and territories in Australia: Australian Capital Territory *n* = 2 (0.5%); New South Wales *n* = 100 (26.5%); Northern Territory *n* = 10 (2.6%); Queensland *n* = 67 (17.7%); South Australia *n* = 31 (8.2%); Tasmania *n* = 11 (2.9%); Victoria *n* = 112 (29.6%); Western Australia *n* = 38 (10.1%). Table 2: outlines the proportions of clinicians asking about other smoking and tobacco exposures.

### Logistic Regressions

Table 3 shows the univariate and multivariate logistic regressions which compares the frequency that clinicians enquire about use of cannabis, chewing tobacco, e-cigarettes (with nicotine), and second-hand smoke exposure with the exposures of interest, type of GP or obstetrician and location.

Cannabis (Table 3): There was a statistically significant univariate association between enquiring about Cannabis and type of clinician (*p* = 0.03); compared to NFATSIH GPs, the RANZCOG GPs (OR = 0.33) and OBS (OR = 0.4) were both significantly less likely to enquire about cannabis (*p* = 0.03). Additionally, there was a statistically significant association between location of practice and enquiring about Cannabis use (*p* = 0.04); compared to practitioners at urban practices, those at regional practices (OR = 1.8) had a higher odds of enquiring about cannabis use (*p* = 0.04). In the multivariable adjusted models, only type of GP (*p* = 0.01) remained statistically significant; the odds of enquiring about Cannabis for RANZCOG GPs was 0.34 lower than compared to NFATSIH GPs.

Chewing Tobacco (Table 3): The univariate model shows that there was a positive association between the remote practitioners and their frequency of asking about a patients use of chewing tobacco (OR = 5.67), however, overall the practice location was not significant. This effect was observed after adjusting for potential confounders, the multivariable model shows that the odds of a remote GP asking about chewing tobacco is 5.02 times higher than an urban GP, again the global *p*-value suggests that the overall effect of location was not significant.

E-cigarettes with nicotine (Table 3): Both the univariate and multivariable models failed to indicate any significant association between the clinician group or location reporting and frequency of enquiry about e-cigarettes. 

Second-Hand Smoke (Table 3): There was a significant association the type of clinician and the frequency they ask about SHS. The univariate model shows the RANZCOG GPs (OR = 0.39) and RANZCOG OBS (OR = 0.23) were significantly (*P* < 0.001) less likely to ask a patient about second hand smoke (SHS) compared to NFATSIH GPs. The multivariate model failed to retain any significant associations between type of clinician or practice location, this suggests that the crude estimates were confounded by demographic differences within the study sample. 

## 4. Discussion

Australian General Practitioners and obstetricians reliably ask pregnant women about cigarette smoking, but less reliably ask about or screen for other exposures, such as cannabis smoking, e-cigarettes, chewing tobacco and second-hand smoke. 

This was a cross-sectional national survey of 378 Australian GPs and obstetricians which analysed the reported frequency of enquiry about other types of smoking and exposures such as cannabis, nicotine through e-cigarettes, chewing tobacco, and second-hand smoke. Compared with the high percentages of these GPs and obstetricians (95%) asking pregnant women “Often-Always” about tobacco smoking, reported previously [5], much fewer “Often-Always” asked about smoking other substances. Overall asking “Often-Always” progressively declined from cannabis (58%), cannabis with tobacco (38%), second-hand smoke (27%), e-cigarettes with nicotine (14%), e-cigarettes without nicotine (13%), and chewing tobacco (10%). The logistic regression showed variable associations with clinician type and the different exposures during pregnancy. Once controlling for other variables, the GPs responding to the online survey who belonged to the NFATSIH performed better than their GP and OBS colleagues from RANZCOG only in asking about cannabis. 

### 4.1. Strengths and Weaknesses of the Study

This is the first study in Australia, as far as we know, to explore whether doctors ask about these other products that may be smoked or consumed. One strength of this study was that it was a national survey, with GPs and Obstetricians from all states and territories in Australia, covering urban, regional and remote areas. Data from RANZCOG indicates that 60% of their members are female, almost identical to our sample of RANZCOG respondents, and RACGP NFATSIH have a somewhat lower representation of female members at 53%, compared to 62% of our respondents.

The low response rates are a limitation, impacting on generalizability [5]. However, surveys of medical practitioners may be less likely to be biased by low response rates. Participants are likely to represent those who might be more interested in this topic and be more likely to respond, thus the findings might represent higher estimates than what is practiced in general. The direction of bias is therefore likely to over-estimate prevalence. Thus, we feel that the prevalence of asking about these substances are conservative. The regressions are unlikely to be biased as the odd ratios are insensitive to prevalence. The inflated prevalence could reduce the power of the log regressions, so therefore any significant associations are less likely to be Type 1 errors.

The survey relied on self-report, so social desirability bias cannot be excluded. We did not ask about the use of hookah or other non-combustible forms of tobacco. We thus advise caution in interpretation on generalizability and representativeness due to these factors.

### 4.2. Strengths and Weaknesses in Relation to other Studies

There have been few similar studies. In a 2012 US study, 53% of 252 obstetricians and gynaecologists reported screening pregnant women at intake for noncombustible tobacco product use, “all or some of the time” [29]. The survey asked these about products as one group and included: chewing tobacco, snuff/snus, e-cigarettes, and dissolvables, thus not differentiating between forms [29]. In another study, only 14% of 776 US paediatricians and family medicine physicians reported screening adolescents for e-cigarette use, compared to 86% screening for tobacco use [30].

### 4.3. Meaning of the Study

The RACGP has published comprehensive guidelines for smoking cessation, last updated in 2014 [31]. They devote separate sections to smoking in pregnancy, Aboriginal and Torres Strait Islanders and culturally and linguistically diverse populations. RANZCOG published a “Women and Smoking” Statement as a literature review to provide advice to members about the management of smoking cessation in pregnancy, last updated in 2014 [32]. Neither guideline covers how clinicians can ask about these other exposures, although the RACGP guidelines mentions the danger of SHS in pregnancy, and mentions chewing tobacco in reference to the Burmese community, but not Aboriginal or Torres Strait Islanders, nor reference to traditional, wild tobacco or *pituri* use [33]. E-cigarettes are mentioned by the RACGP as an unproven method for smoking cessation. 

Currently in Australia, the sale and possession or use of electronic cigarettes containing nicotine is unlawful [34]. Individuals can import nicotine liquid for personal use if a doctor writes a prescription. The Government, on 4 August 2016, called for public debate on exempting low dose nicotine liquid (3.6% or less) for e-cigarette use from their Schedule 7 regulation as a “dangerous poison” [35]. In their interim report, the Therapeutic Goods Authority (TGA) declined to change the listing, and this position is currently being contested by Australian tobacco control experts and researchers [36]. If nicotine liquid is approved in future by the TGA, this would allow wider availability for pregnant women.

### 4.4. Implications for Clinicians or Policymakers

Asking about and recording e-cigarette use, cannabis and SHS may enable monitoring of women and newborns for adverse effects, and be an entry point to advising women to reduce their exposures. After asking about tobacco, women could be asked: “What about any other types of smoking, use of e-cigarettes, cannabis or other ways of using tobacco? Do people smoke inside your home?” Changing provider practices to ask about these other exposures may take time. Inclusion of these other exposures in college and policy guidelines would be a first step, as would including these questions as part of routine antenatal care. Doctors may need training in how to ask about these other smoking, nicotine, vaping and tobacco exposures to ensure women are able to get assistance to reduce potential risks in pregnancy.

### 4.5. Unanswered Questions and Future Research

A more representative sample of clinicians, perhaps including other antenatal staff, would give a more accurate picture of frequency of screening for these products in pregnancy. If this could become a routine part of practice, then important exposures can be tracked and women offered timely advice and assistance to quit smoking, tobacco and nicotine use in all forms. Abstinence would be ideal, as exposure to these substances is not considered desirable during pregnancy for the health of mother and child. 

## 5. Conclusions

A cross-sectional national survey of 378 Australian GPs and obstetricians analysed the reported frequency of enquiry about other types of smoking and exposures such as cannabis, nicotine through e-cigarettes, chewing tobacco, and second-hand smoke. Compared with the high percentages of these GPs and obstetricians (95%) asking pregnant women “Often-Always” about tobacco smoking, much fewer “Often-Always” asked about exposures to or using these other substances. Doctors may need training in how to ask about these other smoking, nicotine, vaping and tobacco exposures to ensure women are able to get assistance to reduce potential risks in pregnancy.

## Figures and Tables

**Table 1:** Demographic characteristics of 378 Australian GP and Obstetrician respondents of a cross-sectional national survey.

Variable	Online Survey	Paper Survey		Total
	NFATSIH GP (*n* = 42) *n* (%)	RANZCOG GP (*n* = 157) *n* (%)	RANZCOG OBS (*n* = 178) *n* (%)	*n* = 378 *n* (%)
Male	16 (38.1)	56 (35.7)	70 (39.3)	142 (37.7)
Female	26 (61.9)	101 (64.3)	108 (60.7)	235 (62.3)
**Smoking Status**				
Current smoker	0 (0)	1 (0.6)	6 (3.4)	7 (1.9)
Ex-smoker	5 (11.9)	21 (13.4)	31 (17.4)	57 (15.1)
Never smoked	37 (88.1)	135 (86)	141 (79.2)	313 (83)
**Years since Medical Qualification**				
<10	13 (31)	21 (13.4)	40 (22.5)	74 (19.6)
10–19	11 (26.2)	39 (24.8)	43 (24.2)	93 (24.7)
20 plus	18 (42.9)	97 (61.8)	95 (53.4)	210 (55.7)
**ASGC-RA Classification of Main Work Location**				
Urban RA1	17 (40.5)	78 (51)	139 (79)	234 (63.1)
Regional RA2 + 3	20 (47.6)	64 (41.8)	33 (18.8)	117 (31.5)
Remote RA 4 + 5	5 (11.9)	11 (7.2)	4 (2.3)	20 (5.4)
**Population Constituency**				
General Population	27 (71.1)	142 (90.4)	175 (98.3)	344 (92.2)
>30% Aboriginal or Torres Strait Islander	11 (28.9)	15 (9.6)	3 (1.7)	29 (7.8)

Legend: RANZCOG Royal Australian and New Zealand College of Obstetricians and Gynaecologists; NFATSIH National Faculty of Aboriginal and Torres Strait Islander Health of Royal Australian College of General Practitioners; GPs General Practitioners; OBS Obstetricians; ASGC-RA Australian Standard Geographical Classification-Remoteness Area.

**Table 2:** Proportions of GPs and Obstetricians who ask about exposures to tobacco, cannabis, chewing tobacco, e-cigarettes, and Second-Hand Smoke.

Variable	NFATSIH GP *n* (%)	RANZCOG GP *n* (%)	RANZCOG OBS *n* (%)	Total *n* (%)
**Tobacco** * (5 missing)				
often-always	40 (97.6%)	151 (96.8%)	164 (93.2%)	355 (95.2%)
never-sometimes	1 (2.4%)	5 (3.2%)	12 (6.8%)	18 (4.8%)
**Cannabis** (20 missing)				
often-always	30 (76.9%)	78 (52.7%)	98 (57.3%)	206 (57.5%)
never-sometimes	9 (23.1%)	70 (47.3%)	73 (42.7%)	152 (42.5%)
**Cannabis with tobacco** (23 missing)				
often-always	23 (59%)	55 (37.7%)	56 (32.9%)	134 (37.7%)
never-sometimes	16 (41%)	91 (62.3%)	114 (67.1%)	221 (62.3%)
**Chewing Tobacco** (23 missing)				
often-always	6 (15.4%)	13 (8.9%)	16 (9.4%)	35 (9.9%)
never-sometimes	33 (84.6%)	133 (91.1%)	154 (90.6%)	320 (90.1%)
**E-Cigarette (nicotine)** (23 missing)				
often-always	8 (20.5%)	22 (15.0%)	20 (11.8%)	50 (14.1%)
never-sometimes	31 (79.5%)	125 (85.0%)	149 (88.2%)	305 (85.9%)
**E-Cigarette (no nicotine)** (24 missing)				
often-always	8 (20.5%)	20 (13.6%)	18 (10.7%)	46 (13%)
never-sometimes	31 (79.5%)	127 (86.4%)	150 (89.3%)	308 (87%)
**Second-Hand Smoke** (21 missing)				
often-always	20 (51.3%)	43 (29.1%)	33 (19.3%)	96 (26.8%)
never-sometimes	19 (48.7%)	105 (70.9%)	138 (80.7%)	262 (73.2%)

Legend: RANZCOG Royal Australian and New Zealand College of Obstetricians and Gynaecologists; NFATSIH National Faculty of Aboriginal and Torres Strait Islander Health of Royal Australian College of General Practitioners; GPs General Practitioners; OBS Obstetricians; * From same sample reported in Bar-Zeev Y. et al. [5].

**Table 3:** Associations between clinician groups, and AGSA-RA, and exposures to cannabis (without tobacco), chewing tobacco, e-cigarettes (with nicotine), and second-hand smoke, in 378 Australian GPs and Obstetricians.

Variable		Cannabis Crude	Cannabis Adjusted ^†^		Chewing Crude	Chewing Adjusted ^†^		E-Cigarette Crude	E-Cigarette Adjusted ^†^		SHS Crude	SHS Adjusted ^†^	
		OR	OR	*p* Value	OR	OR	*p* Value	OR	OR	*p* Value	OR	OR	*p* Value
		(95% CI)	(95% CI)		(95% CI)	(95% CI)		(95% CI)	(95% CI)		(95% CI)	(95% CI)	
**Clinician group**	NFATISH *	1	1	0.01	1	1	0.548	1	1	0.398	1	1	0.064
	RANZCOG GP	0.33	0.34		0.54	0.56		0.68	0.52		0.39	0.49	
		(0.15, 0.75)	(0.13, 0.85)		(0.19, 1.52)	(0.17, 1.8)		(0.28, 1.68)	(0.21, 1.29)		(0.19, 0.8)	(0.22, 1.11)	
	RANZCOG OBS	0.4	0.63		0.57	0.79		0.64	0.5		0.23	0.37	
		(0.18, 0.9)	(0.25, 1.62)		(0.21, 1.57)	(0.24, 2.62)		(0.24, 1.74)	(0.18, 1.39)		(0.11, 0.47)	(0.16, 0.85)	
**Location**	Urban RA1 *	1	1	0.103	1	1	0.077	1	1	0.833	1	1	0.406
	Regional RA2 + RA3	1.8	1.77		1.13	1.09		1.03	0.87		1.17	0.82	
		(1.12, 2.88)	(1.04, 2.99)		(0.5, 2.55)	(0.46, 2.58)		(0.54, 1.98)	(0.43, 1.74)		(0.7, 1.97)	(0.46, 1.45)	
	Remote RA 4 + RA5	1.84	1.06		5.67	5.02		0.77	0.56		5.04	1.87	
		(0.67, 5.09)	(0.28, 4.05)		(1.9, 16.89)	(1.21, 20.8)		(0.17, 3.5)	(0.06, 5.21)		(1.86, 13.65)	(0.53, 6.54)	

Legend: RANZCOG Royal Australian and New Zealand College of Obstetricians and Gynaecologists; NFATSIH National Faculty of Aboriginal and Torres Strait Islander Health of Royal Australian College of General Practitioners; GPs General Practitioners; OBS Obstetricians; SHS second hand smoke; Reference group * NFATSIH GPs, Urban RA1; Note: *p*-values relate to the global significance of the relevant categorical variable, for brevity only the global p-values for the multivariate models are reported. The confidence interval of the OR better represents the significance of the estimate. ^†^ Model adjusted for type of Clinician group, reading of clinical guidelines, location of practice, gender of professional and population constituency.

## References

[1] Hofhuis W., de Jongste J.C., Merkus P.J.F.M. (2003). Adverse health effects of prenatal and postnatal tobacco smoke exposure on children. Arch. Dis. Child..

[2] Australian Institute of Health and Welfare (2015). Australia’s Mothers and Babies 2013—In Brief.

[3] World Health Organization (2013). Who Recommendations for the Prevention and Management of Tobacco Use and Second-Hand Smoke Exposure in Pregnancy.

[4] Passey M.E., D’Este C.A., Sanson-Fisher R.W. (2012). Knowledge, attitudes and other factors associated with assessment of tobacco smoking among pregnant Aboriginal women by health care providers: A cross-sectional survey. BMC Public Health.

[5] Bar-Zeev Y., Bonevski B., Twyman L., Watt K., Atkins L., Palazzi K., Oldmeadow C., Gould G.S. (2017). Opportunities missed: A cross-sectional survey of the provision of smoking cessation care to pregnant women by Australian General Practitioners and Obstetricians. Nicotine Tob. Res..

[6] Juárez S.P., Merlo J. (2013). The effect of Swedish snuff (snus) on offspring birthweight: A sibling analysis. PLoS ONE.

[7] Aslam H.M., Saleem S., German S., Qureshi W.A. (2014). Harmful effects of shisha: Literature review. Int. Arch. Med..

[8] Suliankatchi R.A., Sinha D.N. (2016). The human cost of tobacco chewing among pregnant women in india: A systematic review and meta-analysis. J. Obstet. Gynecol. India.

[9] Sisler L., Meernik C., Ripley-Moffitt C., Greyber J., Goldstein A.O. (2017). Case study: Use of electronic nicotine delivery systems (ENDS) by a pregnant woman. J. Smok. Cessat..

[10] Fallin A., Miller A., Assef S., Ashford K. (2016). Perceptions of Electronic Cigarettes among Medicaid-Eligible Pregnant and Postpartum Women. J. Obstet. Gynecol. Neonatal Nurs..

[11] Mark K.S., Farquhar B., Chisolm M.S., Coleman-Cowger V.H., Terplan M. (2015). Knowledge, attitudes, and practice of electronic cigarette use among pregnant women. J. Addict. Med..

[12] Baeza-Loya S., Viswanath H., Carter A., Molfese D.L., Velasquez K.M., Baldwin P.R., Thompson-Lake D.G.Y., Sharp C., Fowler J.C., De La Garza R. (2014). Perceptions about e-cigarette safety may lead to e-smoking during pregnancy. Bull. Menn. Clin..

[13] England L.J., Bunnell R.E., Pechacek T.F., Tong V.T., McAfee T.A. (2015). Nicotine and the developing human: A neglected element in the electronic cigarette debate. Am. J. Prev. Med..

[14] Spindel E.R., McEvoy C.T. (2016). The Role of Nicotine in the Effects of Maternal Smoking during Pregnancy on Lung Development and Childhood Respiratory Disease. Implications for Dangers of E-Cigarettes. Am. J. Respir. Crit. Care Med..

[15] Lauterstein D.E., Tijerina P., Corbett K., Gordon T., Aschner M., Parmalee N., Conrad K., Allen J., Cory-Slechta D., Zelikoff J.T. (2016). E-cigarette aerosols induce neurobiological and neurobehavioral alterations in mice exposed during early life. [meeting abstract]. Environ. Mol. Mutagen..

[16] Royal College of Physicians (2016). Nicotine without Smoke: Tobacco Harm Reduction.

[17] Hayatbakhsh M.R., Flenady V.J., Gibbons K.S., Kingsbury A.M., Hurrion E., Mamun A.A., Najman J.M. (2011). Birth outcomes associated with cannabis use before and during pregnancy. Pediatr. Res..

[18] Australian Institute of Health and Welfare (2011). 2010 National Drug Strategy Household Survey Report.

[19] Passey M.E., Sanson-Fisher R.W., D’Este C.A., Stirling J.M. (2014). Tobacco, alcohol and cannabis use during pregnancy: Clustering of risks. Drug Alcohol Depend..

[20] Brown S.J., Mensah F.K., Ah Kit J., Stuart-Butler D., Glover K., Leane C., Weetra D., Gartland D., Newbury J., Yelland J. (2016). Use of cannabis during pregnancy and birth outcomes in an aboriginal birth cohort: A cross-sectional, population-based study. BMJ Open.

[21] Gunn J.K.L., Rosales C.B., Center K.E., Nuñez A., Gibson S.J., Christ C., Ehiri J.E. (2016). Prenatal exposure to cannabis and maternal and child health outcomes: A systematic review and meta-analysis. BMJ Open.

[22] Ion R.C., Wills A.K., Bernal A.L. (2015). Environmental tobacco smoke exposure in pregnancy is associated with earlier delivery and reduced birth weight. Reprod. Sci..

[23] Hyland A., Piazza K.M., Hovey K.M., Ockene J.K., Andrews C.A., Rivard C., Wactawski-Wende J. (2014). Associations of lifetime active and passive smoking with spontaneous abortion, stillbirth and tubal ectopic pregnancy: A cross-sectional analysis of historical data from the Women’s Health Initiative. Tob. Control.

[24] Kaufman F.L., Kharrazi M., Delorenze G.N., Eskenazi B., Bernert J.T. (2002). Estimation of environmental tobacco smoke exposure during pregnancy using a single question on household smokers versus serum cotinine. J. Expo. Anal. Environ. Epidemiol..

[25] Kumar S.B., Chawla B., Bisht S., Yadav R.K., Dada R. (2015). Tobacco use increases oxidative DNA damage in sperm—Possible etiology of childhood cancer. Asian Pac. J. Cancer Prev..

[26] Suter M.A., Mastrobattista J., Sachs M., Aagaard K. (2014). Is there evidence for potential harm of electronic cigarette use in pregnancy?. Birth Defects Res. Part A Clin. Mol. Teratol..

[27] Okoli C.T.C., Greaves L., Bottorff J.L., Marcellus L.M. (2010). Health Care providers’ engagement in smoking cessation with pregnant smokers. J. Obstet. Gynecol. Neonatal Nurs..

[28] Australian Government Department of Health Australian Standard Geographical Classification—Remoteness Area (ASGC-RA 2006). http://www.health.gov.au/internet/otd/publishing.nsf/content/ra-intro.

[29] England L.J., Anderson B.L., Tong V.T.K., Mahoney J., Coleman-Cowger V.H., Melstrom P., Schulkin J. (2014). Screening practices and attitudes of obstetricians-gynecologists toward new and emerging tobacco products. Am. J. Obstet. Gynecol..

[30] Pepper J.K., Gilkey M.B., Brewer N.T. (2015). Physicians’ counseling of adolescents regarding e-cigarette use. J. Adolesc. Health.

[31] Zwar N., Richmond R., Borland R., Peters M., Litt J., Bell J., Caldwell B., Ferretter I. (2011). Supporting Smoking Cessation: A Guide for Health Professionals.

[32] The Royal Australian and New Zealand College of Obstetricians and Gynaecologists (2014). Women and Smoking.

[33] Ratsch A., Steadman K.J., Bogossian F. (2010). The pituri story: A review of the historical literature surrounding traditional Australian Aboriginal use of nicotine in Central Australia. J. Ethnobiol. Ethnomed..

[34] Cancer Council Australia & Heart Foundation Position Statement—Electronic Cigarettes. http://wiki.cancer.org.au/policy/Position_statement_-_Electronic_cigarettes.

[35] Australian Government Department of Health—Therapeutic Goods Adminstration Consultation: Proposed Amendments to the Poisons Standard—Acms Meeting, November 2016. https://www.tga.gov.au/consultation-invitation/consultation-proposed-amendments-poisons-standard-acms-meeting-november-2016.

[36] Baker A., Bonevski B., Bonomo Y., Borland R., Day R., Ezard N., Graham R., Hall W., Helliwell D., Lintzeris N. Response to the Scheduling Delegates’ Interim Decision on Nicotine. https://www.clivebates.com/documents/TGAMendelsohn2.pdf.

